# Non-altered incretin secretion in women with impaired fasting plasma glucose in the early stage of pregnancy: a case control study

**DOI:** 10.1186/s13098-023-00981-7

**Published:** 2023-01-30

**Authors:** Ondrej Krystynik, David Karasek, Michal Kahle, Veronika Kubickova, Dominika Macakova, Lubica Cibickova, Milos Mraz, Martin Haluzik

**Affiliations:** 1 Third Department of Internal Medicine – Nephrology, Rheumatology and Endocrinology, University Hospital Olomouc and Faculty of Medicine and Dentistry, Palacky University, I. P. Pavlova 6, 77900 Olomouc, Czech Republic; 2 Department of Data Science, Institute for Clinical and Experimental Medicine, Vídeňská 1958,140 21, Prague, Czech Republic; 3 Department of Clinical Biochemistry, University Hospital Olomouc, I. P. Pavlova 6, 77900 Olomouc, Czech Republic; 4 Diabetes Centre, Institute for Clinical and Experimental Medicine, Vídeňská 1958, 140 21, Prague, Czech Republic

**Keywords:** Gestational diabetes, Glucagon-like peptide 1, Glucose-dependent insulinotropic peptide, Glucagon, Meal test

## Abstract

**Backgrounds:**

Glucagon-like peptide 1 (GLP-1) and glucose-dependent insulinotropic peptide (GIP) may be involved in pathogenesis of gestational diabetes mellitus (GDM). The aim was to compare GLP-1 and GIP production in fasting state and during 3 h mixed meal tolerance test (MMTT) measured by mean area under the curve (AUC) between pregnant women with normal and impaired fasting glucose in an early phase of pregnancy, and healthy non-pregnant controls.

**Methods:**

This study was undertaken as a case–control study. Repeated measurement of fasting plasma glucose ≥ 5.1 mmol/L and < 7.0 mmol/L during the first trimester of pregnancy and exclusion of overt diabetes according to IADSPG criteria was used to find women with impaired fasting glucose (n = 22). Age-matched controls consisted of healthy pregnant (n = 25) and non-pregnant (n = 24) women. In addition to incretins, anthropometric parameters and markers of insulin resistance and beta-cell function were assessed. Variables were summarized as median (interquartile range).

**Results:**

Fasting GLP-1 and GIP concentration or their AUC during MMTT did not significantly differ between pregnant women with impaired fasting plasma glucose [GLP-1_AUC_ 19.0 (53.1) and GIP_AUC_ 302 (100) pg/mL/min] and healthy pregnant women [GLP-1_AUC_ 16.7 (22.3) and GIP_AUC_ 297 (142) pg/mL/min] or non-pregnant controls [GLP-1_AUC_ 16.8 (9.8) and for GIP_AUC_ 313 (98) pg/mL/min]. Although women with impaired fasting glucose were more obese and showed decreased beta-cell function, there were not significant correlations between incretin production and parameters of insulin secretion, insulin resistance, or obesity.

**Conclusions:**

Women with impaired fasting plasma glucose did not show altered incretin production in the first trimester of pregnancy. In contrast to type 2 diabetes, impaired incretin secretion does not seem to play a major role in the early development of GDM.

## Background

Gestational diabetes mellitus (GDM) is defined as glucose intolerance first detected during pregnancy that resolves after delivery. GDM affects 5–30% of pregnancies, depending on the diagnostic criteria used [1–3]. The pathophysiology of GDM is not yet fully understood, although it generally involves relatively insufficient insulin secretion with increased peripheral insulin resistance that develops during pregnancy [4].

Incretins such as glucagon-like peptide 1 (GLP-1) and glucose-dependent insulinotropic peptide (GIP) could be involved in mechanisms compensating the increase in blood glucose due to insulin resistance observed in pregnant women [5]. Both GLP-1 and GIP have additive effects on insulin secretion following their increased production induced by nutrients from the gut [6]. Impaired incretin effect may contribute to impaired glucose postprandial control, which is characteristic of some GDM phenotypes. GLP-1 suppresses, and GIP increases glucagon secretion. Glucagon abnormalities (increased fasting levels and delayed glucagon suppression after glucose intake) has been reported in type 2 diabetes; however, its role in GDM is still unknown. Only a few studies have investigated fasting or postprandial GLP-1, GIP and/or glucagon concentrations in patients with GDM and reported contrasting results [7–14]. These discrepant findings may be due to the variable representation of women with different GDM phenotypes, i.e. with a varied contribution of insulin resistance, impaired postprandial insulin secretion and/or incretin resistance. A different methodology for determining incretin levels, such as different laboratory kits and the use of various stimulating meal compositions, can play a role in the discordant results of postprandial incretin concentrations [15]. Comparative studies have shown that mixed meal tolerance test (MMTT), based on the ingestion of a standardized liquid meal, induces a stronger beta-cell response than the oral glucose tolerance test (OGTT) in the individuals with impaired glucose tolerance and type 2 diabetes [16]. The use of the MMTT probably leads to a greater response of both incretin hormones and according to some authors is better suited to detect small differences in their secretion, which may explain some of the different results also in subjects with GDM [7, 8, 10]. The incretin response may also be dependent on the period of pregnancy. Little is known about altered incretin production in the early phase of gravidity and its potential role in the development of GDM during this period.

According to current recommendations, the first step to diagnose glucose intolerance in pregnancy should be performed during the initial visit (usually in the first trimester) to detect women with overt diabetes who have not been previously diagnosed outside of pregnancy [17]. However, there is still debate over the correct diagnostic criteria for GDM in the first part of pregnancy. The International Association of Diabetes and Pregnancy Study Groups (IADPSG) recommended to classify fasting plasma glucose (FPG) ≥ 5.1 mmol/L and < 7.0 mmol/L as early GDM [18]. Unfortunately, these recommendations were not derived from sufficient clinical data obtained in early pregnancy and have therefore been criticized. Recently, it has been recommended to identify women with FPG > 6.1 mmol/L in early pregnancy because of the increased risk of developing GDM later in pregnancy and the increased risk of pregnancy and neonatal complications [17]. On the one hand, the IADPSG recommendations for early screening and detection of GDM are not based on sufficient clinical data, but on the other hand, application of the IADPSG early pregnancy screening process has been shown to identify women at increased risk of elevated blood pressure and insulin resistance in later gestation [19]. Impaired incretin effect may occur early in the disease process, as shown in adults and adolescents with insulin resistance and obesity [20, 21]. Thus, we can hypothesize that women with impaired fasting glucose in early pregnancy compared to pregnant women with normal glucose tolerance will show an altered incretin response.

The primary aim of our study was to investigate whether there is a difference in GLP-1, GIP, and glucagon response during a 3-h oral MMTT measured by total area under the curve (AUC) between pregnant women with impaired FPG in the first trimester of pregnancy compared to control pregnant and non-pregnant subjects. Secondary aim was to examine the relationship between incretin production and parameters of insulin resistance and beta-cell function.

## Material and methods

### Study design, inclusion and exclusion criteria

The study was undertaken as a case–control study in accordance with the principles of the Declaration of Helsinki as revised in 2008. It was reviewed and approved by the Ethics Committee of Medical Faculty and University Hospital Olomouc (approval no. 120/17) and informed consent was obtained from all participants. Pregnant women were eligible for recruitment to the study if they were within 8–14 weeks of pregnancy. All participants were asked about their personal and medical history. Weight and height were measured at the first antenatal visit (1st trimester of pregnancy), the body mass index (BMI) was calculated as body weight/body height^2^ (kg/m^2^). The diagnosis of early impairment of fasting plasma glucose (i-FPG) was based on at least two measurements of fasting plasma glucose ≥ 5.1 mmol/L and < 7.0 mmol/L on two different days during the first trimester (8.-14. week) of pregnancy [17, 18]. The exclusion criteria were: type 1 or type 2 diabetes, secondary or genetic type of diabetes, a history of GDM, renal, liver or thyroid disease, drug or alcohol abuse and concomitant medication affecting glucose metabolism and GIT function (glucocorticoids, proton pump inhibitors, prokinetics, pancreatic enzyme products). Healthy pregnant women had normal glucose levels throughout their pregnancy, including the subsequent OGTT at 24–28 weeks of gestation [17, 18]. Non-pregnant age-matched controls comprised healthy women without a personal history of glucose intolerance or diabetes (including GDM or a history of delivering a high birth weight baby, i.e. ≥ 4.5 kg).

### Mixed meal tolerance test

Each MMTT was performed with standardized liquid meals after 10 h overnight fasting, in pregnant women during the first trimester (8–14 week) of pregnancy. Blood samples were collected at baseline and at 30, 60, 120, and 180 min after ingestion of 200 ml of commercially available liquid enteral nutrition (Fresubin Original Drink^®^, Fresenius Kabi, Germany; 100 ml contains 420 kJ of energy intake, i.e. 3.4 g (30 kJ %) of fat, 13.8 g (55 kJ %) of carbohydrates, and 3.8 g (15 kJ %) of proteins) between 7 and 10 am. Samples for measurement of GLP-1, GIP, and glucagon were collected into special blood tubes with an inhibitor cocktail consisting of a dipeptidyl peptidase 4, esterase and other protease inhibitors (BD P800, Becton, Dickinson and Company, Franklin Lakes, USA) and then separated by centrifugation for 10 min at 1000 g within 30 min of blood collection. Plasma was subsequently stored in aliquots at − 80 °C until further analysis.

### Laboratory analyses

Routine biochemical parameters (glucose, HbA1C, insulin, C-peptide) were measured by an automated analyzer Cobas 8000 (Roche) on the day of blood collection. Concentrations of incretins and glucagon were measured in sample aliquots stored at − 80 °C for no longer than 6 months.

Glucose levels were determined using the hexokinase method (GLUC3, Roche Diagnostics GmbH, Mannheim, Germany). Levels of HbA1C were measured by ion exchange chromatography using the Arkray Adams HA-8180 V analyzer (Arkray Corporation, Kyoto, Japan). Insulin and C-peptide levels were determined with commercially available kits (Immunotech, Marseille, France) using an immunoradiometric assay with specific antibodies. Based on fasting glucose and insulin levels the homeostasis model assessment of β-cell function (HOMA-β), homeostasis model assessment of insulin resistance (HOMA-IR) indexes [22], and quantitative insulin sensitivity check index (QUICKI) [23] were calculated.

Plasma glucagon, GLP-1, and GIP concentrations were measured by commercial multiplex assay (Human Metabolic Hormone Magnetic Bead Panel, HMHEMAG- 34 K, Merck Millipore, USA). Sensitivity was 13.0 pg/mL for glucagon, 1.2 pg/mL for GLP-1 and 0.6 pg/mL for GIP. Intra- and inter-assay variabilities for the kits were < 10% and 15%, respectively.

### Statistical analyses

All variables are summarized as mean ± standard deviation (SD) for normally distributed data or median (interquartile range) otherwise. Normal distribution was tested by Shapiro–Wilk’s test. Differences in variables between the groups were analyzed with the t-test for normally distributed variables or with the Mann–Whitney U-test for non-normally distributed variables. Spearman coefficient (ρ) was used to express the value of correlation. The mean area under the curve (AUC) was calculated using the trapezoidal rule divided by the time interval. The distribution of GIP_AUC_ is approximately normal. Given the distribution of the measurements and the desired power of 80% we would need 16 participants in each group to detect a difference of 33% (i.e. 100 pg/mL) in any direction by a two-tailed t-test. The distribution of GLP-1_AUC_ is approximately normal after log transformation. Given the distribution of the measurements and the desired power of 80% we would need 26 participants in each group to detect an increase of 100% or decrease of 50% in the original scale by a two-tailed t-test. *P* < 0.05 was considered as significant. Statistical analyses were performed using the Python ecosystem.

## Results

### Basic clinical and laboratory characteristic and incretins levels in individual groups

**Fig. 1:**
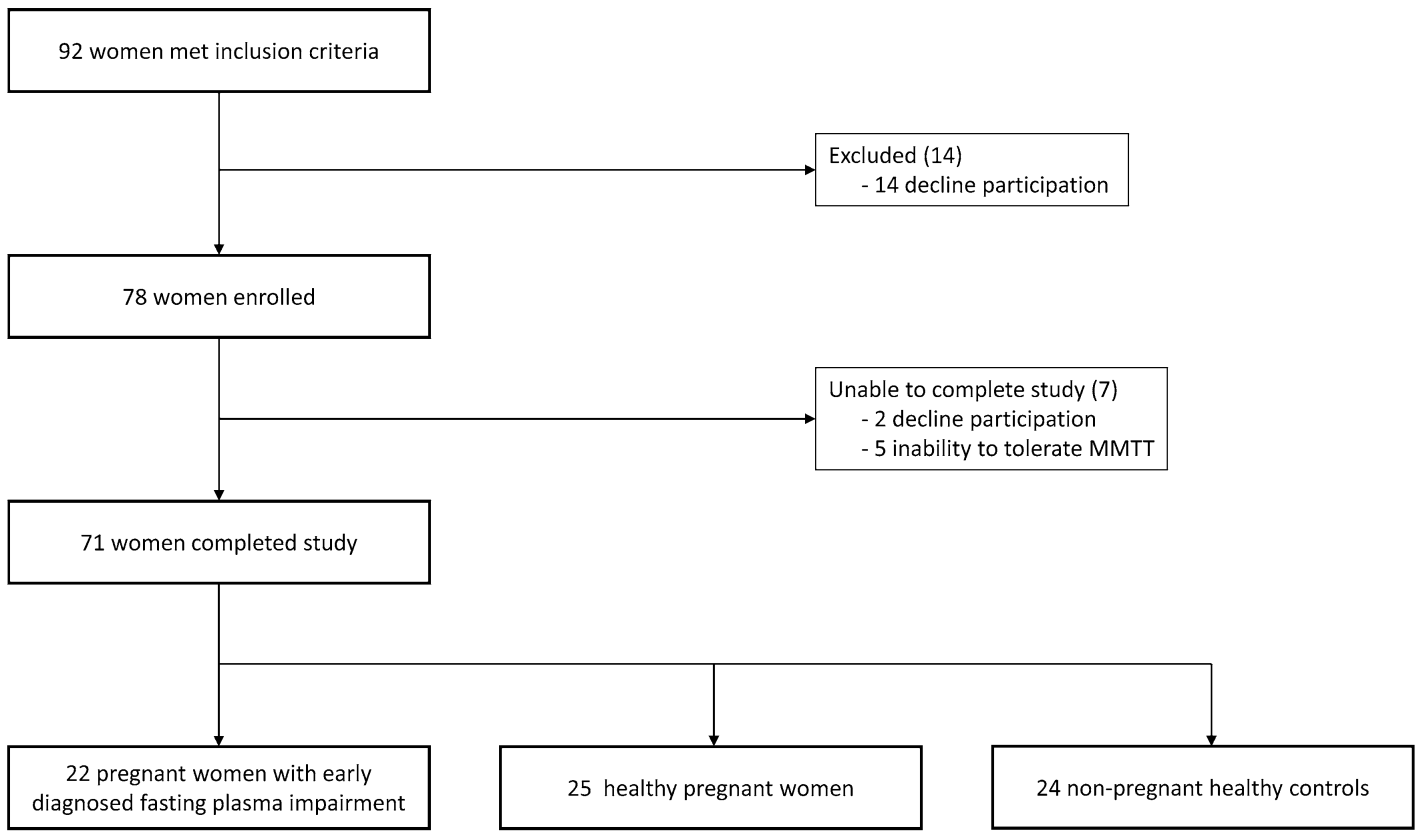
Flow diagram of study participants, *MMTT* mixed meal tolerance test

**Fig. 2:**
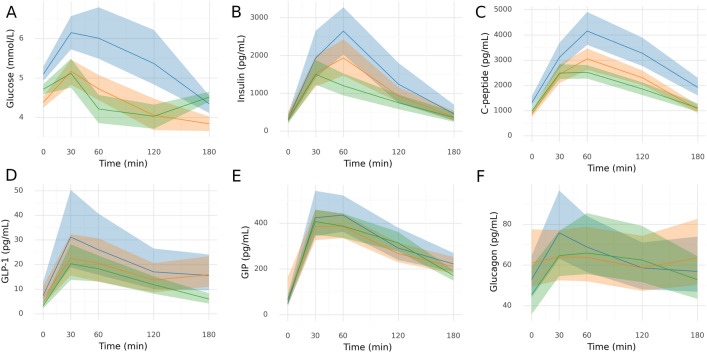
Changes of glucose and hormones levels during MMTT in individual groups. Mean and 95% confidence intervals are shown for glucose, GIP and glucagon. Insulin, C-peptide and GLP-1 follow approximately log-normal distribution so geometric mean (equivalent to arithmetic mean in log transformed data) and 95% confidence intervals are displayed. Confidence intervals were obtained in all cases by 10,000 times bootstrapping. Blue–for women with impaired fasting plasma glucose, red—for healthy pregnant women, green – for non-pregnant healthy controls

Twenty-two pregnant women with early diagnosed fasting plasma impairment, 25 healthy pregnant women and 24 non-pregnant healthy controls met eligible criteria for this study (see Fig. 1 and Table 1). All participants were of Caucasian ethnicity. Women with i-FPG had significantly higher BMI, FPG, HbA_1C_, and C-peptide levels compared to healthy pregnant women or compared to non-pregnant healthy controls. Only in comparison to healthy pregnant women they also had lower HOMA-β. Healthy pregnant women had significantly lower FPG compared to non-pregnant healthy controls. There were no differences between groups in HOMA-IR and QUICKI. FPG and HbA_1C_ levels in all pregnant women positively correlated with BMI (ρ = 0.37, ρ = 0.49 respectively). No significant differences in fasting plasma concentration of GLP-1, GIP, or glucagon between pregnant women were observed at time 0. Compared to non-pregnant healthy controls both pregnant women had lower levels of GIP. In pregnant women with i-FPG there were also significantly higher fasting GLP-1 concentrations compared to non-pregnant controls—see Table 1, Fig. 2.

**Table 1:** Basic clinical and laboratory characteristics in individual groups

	Pregnant women i-FPG (n = 22)	Healthy pregnant women (n = 25)	Non-pregnant healthy controls (n = 24)
age (years)	31.6 ± 5.0	29.4 ± 3.0	29.2 ± 3.5
gestational age (weeks)	11.3 ± 2.1	11.0 ± 1.9	–
Weight (kg)	80.0 ± 14.5^b,c^	68.0 ± 12.3^a^	64.0 ± 6.8^a^
BMI (kg/m^2^)	28.9 ± 5.4^b,c^	23.4 ± 4.0^a^	22.6 ± 2.8^a^
FPG (mmol/L)	5.1 ± 0.4^b,c^	4.4 ± 0.4^a,c^	4.7 ± 0.3^a,b^
HbA_1C_ (mmol/mol)	33.0 ± 2.7^b,c^	29.9 ± 2.5^a^	31.1 ± 1.8^a^
C-peptide (pg/mL)	1211 (720)^b,c^	840 (378)^a^	1078 (526)^a^
Insulin (pg/mL)	348 (381)	306 (343)	296 (324)
HOMA-IR	2.01 (1.85)	1.40 (1.55)	1.59 (1.80)
HOMA-β	105 (108)^b^	214 (283)^a^	173 (143)
QUCKI	0.34 (0.05)	0.36 (0.05)	0.36 (0.05)
GLP-1 (pg/mL)	6.4 (14.9)^c^	4.7 (19.9)	2.6 (5.7)^a^
GIP (pg/mL)	36.3 (28.4)^c^	36.3 (35.5)^c^	56.5 (46.6)^a,b^
Glucagon (pg/mL)	49.2 (31.4)	50.6 (32.2)	38.9 (42.2)

Values are expressed means ± standard deviation or medians and interquartile ranges (for data with non-normal distribution). Laboratory samples were taken in a fasting state. All investigated women represent the Caucasian population

Uncorrected differences (p < 0.05) according to the t-test (for normally distributed variables) or to the Mann–Whitney U-test (for non-normally distributed variables):

*i-FPG* impaired fasting plasma glucose, *BMI* body mass index, *FPG* fasting plasma glucose, *HbA*_*1C*_ glycated hemoglobin A_1C_, *HOMA-IR* homeostasis model assessment of insulin resistance, *HOMA-β* homeostasis model assessment of β-cell function, *QUCKI*  quantitative insulin sensitivity check index, *GLP-1*  glucagon-like peptide 1, *GIP* glucose-dependent insulinotropic peptide

^a^ = *vs* pregnant women with Ifpg

^b^ = *vs* healthy pregnant women

^c^ = *vs* non-pregnant healthy controls

### Changes of glucose and hormones levels during MMTT

Figure 2 shows changes in glucose and hormone levels during the MMTT. Women with i-FPG had significantly higher C-peptide levels throughout the MMTT and higher blood glucose levels at 30, 60, and 120 min of the MMTT compared to healthy pregnant or non-pregnant controls. They also had higher insulin levels after 60 and 120 min of the MMMT, but only compared to non-pregnant women. Healthy pregnant women had significantly lower glycemia at 180 min, higher C-peptide at 60 and 120 min, and insulin at 60 min of the MMTT compared with non-pregnant controls. Table 2 shows AUC for glucose and hormones levels in individual groups. Glucose_AUC_, C-peptide_AUC_, and insulin_AUC_ were significantly increased in pregnant women with i-FPG compared to healthy pregnant women or non-pregnant controls. These differences were not significant after adjustment for BMI. Higher GLP-1 levels in all pregnant women in comparison to non-pregnant controls were observed only at 180 min of the MMTT. No other significant differences in GLP-1 were detected between groups during the MMTT test and there were no significant differences in glucagon and GIP levels. No significant differences in GLP-1_AUC_, GIP_AUC_, glucagon_AUC_ between investigated groups were detected – see Table 2.

**Table 2:** Area under the curve during 3 h mixed meal tolerance test for glucose and hormones levels in individual groups

	Pregnant women i-FPG (n = 22)	Healthy pregnant women (n = 25)	Non-pregnant healthy controls (n = 24)	corrected p-value
glucose_AUC_ (mmol/mL/min)	5.47 ± 1.00^b,c^	4.40 ± 0.64^a^	4.40 ± 0.53^a^	< 0.001
glucose_AUC_ adjusted for BMI (mmol/mL/min)	5.05 ± 0.88	4.54 ± 0.73	4.62 ± 0.48	NS
C-peptide_AUC_ (pg/mL/min)	3037 (833)^b,c^	2307 (822)^a^	2092 (554)^a^	< 0.001
C-peptide_AUC_ adjusted for BMI (pg/mL/min)	2716 (954)	2477 (941)	2235 (760)	NS
insulin_AUC_ (pg/mL/min)	1979 (973)^b,c^	1271 (734)^a^	1001 (692)^a^	0.007
insulin_AUC_ adjusted for BMI (pg/mL/min)	1751 (660)	1485 (940)	1079 (723)	NS
GLP-1_AUC_ (pg/mL/min)	19.0 (53.1)	16.7 (22.3)	16.7 (9.8)	NS
GLP-1_AUC_ adjusted for BMI (pg/mL/min)	19.6 (54.9)	16.9 (18.5)	17.0 (10.6)	NS
GIP_AUC_ (pg/mL/min)	317 ± 124	290 ± 89	303 ± 80	NS
GIP_AUC_ adjusted for BMI (pg/mL/min)	336 ± 119	286 ± 85	297 ± 81	NS
glucagon_AUC_ (pg/mL/min)	63.2 ± 28.1	61.7 ± 32.9	60.6 ± 29.5	NS
glucagon_AUC_ adjusted for BMI (pg/mL/min)	65.8 ± 26.9	61.6 ± 32.9	60.3 ± 29.4	NS

Values are expressed means ± standard deviation or medians and interquartile ranges (for data with non-normal distribution). Difference between groups was tested by one-way ANOVA for normally distributed variables or Kruskal–Wallis test otherwise. Holm-Bonferroni procedure was used to correct for multiple testing. In cases of significant difference pairwise tests were performed by t-test for normally distributed variables or to the Mann–Whitney U-test otherwise:

*i-FP * impaired fasting plasma glucose, *GLP-1* = glucagon-like peptide 1, *GIP* glucose-dependent insulinotropic peptide, *AUC* area under the curve during 3 h mixed meal tolerance test, *NS* non-significant

^a^ = *vs* pregnant women with i-FPG

^b^ = *vs* healthy pregnant women

^c^ = *vs* non-pregnant healthy controls

There was a correlation between GLP-1_AUC_ and GIP_AUC_ (ρ = 0.41) and between glucagon_AUC_ and both GLP-1_AUC_ (ρ = 0.37) and GIP_AUC_ (ρ = 0.36) in all pregnant women. In pregnant women with i-FPG, glucagon_AUC_ correlated with GLP-1_AUC_ (ρ = 0.48), whereas in healthy pregnant women there was a correlation between glucagon_AUC_ and GIP_AUC_ (ρ = 0.49). Correlations between selected baseline parameters and GLP-1_AUC_, GIP_AUC_ and glucagon_AUC_ are presented in the Table 3.

**Table 3:** Correlations between baseline parameters and incretins

		iFPG		Healthy pregnant		All Pregnant		Non-pregnant	
		fasting	AUC	fasting	AUC	fasting	AUC	fasting	AUC
Age	GLP-1	− 0.09	− 0.08	0.21	0.23	0.07	0.12	− 0.16	− 0.23
	GIP	− 0.33	− 0.04	0.18	0.14	− 0.08	0.09	− 0.21	− 0.06
	Glucagon	− 0.01	− 0.21	− 0.02	0.09	0.02	-0.04	− 0.26	− 0.20
BMI	GLP-1	− 0.15	− 0.42	− 0.01	− 0.09	0.01	-0.12	− 0.42*	− 0.37
	GIP	− 0.35	− 0.08	− 0.01	− 0.36	− 0.17	-0.12	− 0.17	0.08
	Glucagon	0.25	0.10	0.04	− 0.12	0.09	0.09	− 0.34	− 0.27
HbA1c	GLP-1	0.08	0.02	0.14	0.09	0.15	0.10	0.02	− 0.08
	GIP	− 0.51*	− 0.09	− 0.13	− 0.13	− 0.30*	-0.08	− 0.15	− 0.11
	Glucagon	0.30	0.17	0.08	− 0.07	0.11	0.03	− 0.37	− 0.28
FPG	GLP-1	− 0.08	0.02	− 0.47*	− 0.15	− 0.19	0.03	0.10	− 0.14
	GIP	− 0.43*	− 0.16	− 0.50*	− 0.27	− 0.36*	-0.13	0.02	0.19
	Glucagon	0.26	0.15	− 0.40*	− 0.44 *	− 0.16	-0.09	− 0.11	− 0.06
C-peptide	GLP-1	0.06	− 0.13	0.40*	0.20	0.24	0.05	− 0.02	0.03
	GIP	0.02	− 0.05	0.17	0.41 *	0.08	0.21	0.39	0.37
	Glucagon	0.05	− 0.01	0.17	0.10	0.08	0.06	0.41*	0.45*
HOMA-IR	GLP-1	0.27	0.12	0.30	0.03	0.29	0.12	− 0.08	0.04
	GIP	− 0.18	0.16	0.42*	0.14	0.15	0.15	0.17	− 0.02
	Glucagon	0.34	0.29	0.32	0.26	0.33*	0.27	0.24	0.39
HOMA-β	GLP-1	0.30	0.13	0.45*	0.05	0.35*	0.08	− 0.08	0.03
	GIP	− 0.06	0.11	0.66***	0.33	0.36*	0.20	0.27	− 0.04
	Glucagon	0.25	0.23	0.45*	0.45 *	0.42**	0.33*	0.36	0.45*
QUICKI	GLP-1	− 0.27	− 0.12	− 0.30	− 0.03	− 0.29	− 0.12	0.08	− 0.04
	GIP	0.18	− 0.16	− 0.42*	− 0.14	− 0.15	− 0.15	− 0.17	0.02
	Glucagon	− 0.34	− 0.29	− 0.32	– 0.26	− 0.33*	− 0.27	− 0.24	− 0.39

*iFPG * impaired fasting plasma glucose, *BMI* body mass index, *FPG*  fasting plasma glucose, *HbA*_*1C*_ glycated hemoglobin A_1C_, *HOMA-IR* homeostasis model assessment of insulin resistance, *HOMA-β *homeostasis model assessment of β-cell function, *QUCKI* quantitative insulin sensitivity check index, *GLP-1* glucagon-like peptide 1, *GIP* glucose-dependent insulinotropic peptide, *AUC* area under the curve during 3-h mixed meal tolerance test

For fasting levels and AUC mean of GLP-1, GIP and glucagon were correlated with baseline factors using Spearman coefficient (ρ). Stars denote uncorrected statistical significance

^*^p < 0.05

^**^p < 0.01

^***^p < 0.001

## Discussion

Studies focused on the possible role of incretin hormones in the development of gestational diabetes mellitus have brought conflicting results. Here, we tested the hypothesis that women with impaired fasting glucose will demonstrate an altered incretin response during mixed meal tolerance test in an early phase of pregnancy as compared to pregnant women with normal glucose tolerance. As expected, women with early diagnosed i-FPG were more obese and showed signs of decreased beta-cell function measured by HOMA-β. Although they had higher C-peptide levels, markers of insulin resistance (HOMA-IR, QUICKI) did not significantly differ compared to controls. Despite the impaired glucose homeostasis and insulin secretion, our study did not find significant differences in GLP-1, and GIP concentration during fasting state or during 3-h oral MMTT (measured as AUC) between pregnant women with impaired fasting glucose diagnosed in the first trimester of pregnancy and age-matched healthy pregnant controls.

Only a few studies investigated GLP-1 and GIP concentrations in women with GDM and reported contrasting results. Cypryk et al. did not find an impaired secretion of GLP-1 and GIP during OGTT in women with GDM [7]. They were more insulin resistant compared to controls. Decreased GLP-1 secretion during OGTT in women with GDM was observed by Lencioni et al. [8]. These women had similar weight and BMI compared to the healthy pregnant controls, but they showed lower first-phase insulin secretion and insulin sensitivity secretion index. Reduced GLP-1 responses during OGTT were also found by Sukumar et al. [9]. These GDM subjects showed impaired insulin secretion, but they did not differ in HOMA-IR. Bonde et al. investigated postprandial GLP-1 responses in women with GDM during the MMTT [10]. They showed reduced postprandial GLP-1 secretion compared to 3–4 months postpartum, but the GLP-1 response did not differ from women with normal glucose tolerance during pregnancy (in the third trimester). Mosavat et al. found lower both GLP-1 and GIP fasting levels in patients with GDM [11]. They also had lower β-cell function as measured by HOMA-β. Lower GLP-1 and GIP were independently associated with a higher risk for GDM. Surprisingly, Fritsche et al. detected increased GLP-1 and GIP secretion during OGTT in women with GDM [12]. These women had lower insulin secretion and similar BMI compared to healthy controls. Authors explain the pronounced GLP-1 response as part of a compensatory mechanism counteracting GLP-1 resistance in GDM subjects. All above mentioned studies focused on women with GDM diagnosed in the second trimester. Discrepant findings may be due to varied proportion of insulin resistance, and/or impaired insulin secretion (due to impaired incretin production or incretin resistance) in women with different GDM phenotypes. In our study, although women with impaired fasting glucose in the first trimester showed decreased beta-cell function, GLP-1 or GIP fasting concentrations were not altered and no significant changes in their secretion during a 3-h oral MMTT were detected. Our study thus does not support the possibility that impaired incretin secretion plays a major role in the development of early GDM.

Similar to others [9, 12] we also did not detect differences in glucagon fasting or postprandial secretion between pregnant women. GLP-1 inhibits, whereas GIP stimulates glucagon secretion [24]. A significant positive correlation between glucagon and both incretin production during MMTT was found in all pregnant women. In healthy pregnant women there was a significant correlation only between glucagon_AUC_ and GIP_AUC_, while in women with impaired fasting glucose glucagon_AUC_ correlated only with GLP-1_AUC_. Inappropriate positive association between glucagon and GLP-1 secretion during MMTT was also detected in C-peptide positive individuals with type 1 diabetes [25]. Whether this finding is clinically significant is debatable. Impaired suppression of glucagon by GLP-1 as a manifestation of incretin resistance may be involved in hyperglycemia [14, 15], however our results argue against a major contribution of increased glucagon production in the pathogenesis of early GDM.

One of the possible reasons for lack of difference in incretin secretion between groups in our study could lie in our focus on pregnant women with early diagnosed i-FPG. These results thus cannot be extrapolated to women with normal glucose levels during the first trimester of pregnancy who are diagnosed with GDM later according to abnormal oGTT at 24–28. week of pregnancy. We did not perform MMTT in later stages of pregnancy, so we cannot exclude the possibility that altered incretin secretion in women with early diagnosed impaired fasting glucose may appear during later course. Another reason for lack of difference in incretin secretion between groups may be the lack of ethnic diversity – all participants were of Caucasian ethnicity. Different racial/ethnic backgrounds are associated with different prevalence of glucose intolerance during pregnancy [5, 17]. The homogenous sample of examined pregnant women may be a limitation of this study.

In contrast to conflicting data on the role of incretins, obesity seems to be more important factor for GDM development. Maternal obesity was associated with higher C-peptide and lower insulin sensitivity even in the first trimester of pregnancy [26]. Diet and early mild-moderate exercise have been effective in GDM prevention [27]. In our study, we found higher levels of C-peptide both in fasting state and during the MMTT in women with i-FPG compared to healthy controls as well. However, after adjustment for BMI C-peptide_AUC_ did not show a significant difference. Although BMI did not correlate with both fasting and postprandial incretin production and women with impaired fasting glucose did not show a significant increase in markers of insulin resistance (HOMA-IR, QUICKI), they had reduced beta-cell function as measured by HOMA-β. Obesity can impair glycemic compensation not only through insulin resistance but also through disruption of beta-cell function due to adverse secretion of adipokines [28, 29]. Unbalanced adipokine (low adiponectin, high leptin) levels were detected even in overweight women with early-onset GDM [30]. Recently, we have also found in women with early GDM altered adipokines production (increased adipocyte fatty acid-binding protein and decreased adiponectin levels) that correlated with visceral adiposity and glucose control. The introduction of lifestyle interventions during pregnancy and early GDM treatment was associated with not only prevention of weight gain and with glycemic compensation, but also normalization of adipokines levels [31]. These findings support the possible role of endocrine dysfunction of adipose tissue in the development of GDM.

## Conclusion

Women with early diagnosed impaired fasting glucose did not show altered fasting or postprandial incretin secretion in the first trimester of pregnancy. These patients were more obese and showed signs of decreased beta-cell function. In contrast to obesity, impaired incretin secretion does not seem to play a major role in the early development of GDM.

## Data Availability

The data used in this manuscript are available upon request.

## Abbreviations

AUC: Area under the curve
BMI: Body mass index
FPG: Fasting plasma glucose
GDM: Gestational diabetes mellitus
GIP: Glucose-dependent insulinotropic peptide
GLP-1: Glucagon-like peptide 1
HbA_1C_: Glycated hemoglobin A_1C_
HOMA-IR: Homeostasis model assessment of insulin resistance
HOMA-β: Homeostasis model assessment of β-cell function
IADPSG: International association of diabetes and pregnancy study groups
i-FPG: Impaired fasting plasma glucose
MMTT: Mixed meal tolerance test
OGTT: Oral glucose tolerance test
QUCKI: Quantitative insulin sensitivity check index
